# Tapping into the power of coproduction and knowledge mobilisation: Exploration of a facilitated interactive group learning approach to support equity‐sensitive decision‐making in local health and care services

**DOI:** 10.1111/hex.13774

**Published:** 2023-05-08

**Authors:** Jane Cloke, Shaima Hassan, Mark Goodall, Adele Ring, Pooja Saini, Naheed Tahir, Mark Gabbay

**Affiliations:** ^1^ Department of Primary Care and Mental Health University of Liverpool Liverpool UK; ^2^ School of Psychology Liverpool John Moores University Liverpool UK; ^3^ NIHR ARC NWC Liverpool UK

**Keywords:** capacity building, coproduction, health inequalities, knowledge mobilisation

## Abstract

**Background:**

We report on a study of a facilitated interactive group learning approach, through Collaborative Implementation Groups (CIGs), established to enhance capacity for equity‐sensitive evaluation of healthcare services to inform local decision‐making: (1) What was the experience of participants of the CIGs? (2) How was knowledge mobilisation achieved? (3) What are the key elements that enhance the process of coproducing equity‐sensitive evaluations?

**Methods:**

A thematic analysis of qualitative data obtained from focus group (FG) discussions and semistructured interviews exploring the experiences of participants. All FGs included representation of participants from different projects across the programme. Interviews were conducted with a member from each of the teams participating in the first cohort after their final workshop.

**Results:**

We identified four themes to illustrate how the approach to delivering intensive and facilitated training supported equity‐sensitive evaluations of local healthcare services: (1) Creating the setting for coproduction and knowledge mobilisation; (2) establishing a common purpose, meaning and language for reducing health inequalities; (3) making connections and brokering relationships and (4) challenging and transforming the role of evaluation.

**Conclusion:**

We report on the implementation of a practical example of engaged scholarship, where teams of healthcare staff were supported with resources, interactive training and methodological advice to evaluate their own services, enabling organisations to assemble timely practical and relevant evidence that could feed directly into local decision‐making. By encouraging mixed teams of practitioners, commissioners, patients, the public and researchers to work together to coproduce their evaluations, the programme also aimed to systematise health equity into service change. The findings of our study illustrate that the approach to delivering training gave participants the tools and confidence to address their organisation's stated aims of reducing health inequalities, coproduce evaluations of their local services and mobilise knowledge from a range of stakeholders.

**Patient or Public Contribution:**

The research question was developed collaboratively with researchers, partner organisations and public advisers (PAs). PAs were involved in meetings to agree on the focus of this research and to plan the analysis. N. T. is a PA and coauthor, contributing to the interpretation of findings and drafting of the paper.

## 1 BACKGROUND/INTRODUCTION

### 1.1 Using research in commissioning and decision‐making in healthcare

Significant investment is made in producing research evidence to inform decisions about the delivery and organisation of healthcare. However, much has been written on the significant delays in putting research into clinical practice, how this impacts patient care^1^ and why researchers and funding bodies must accelerate the process^2^, ^3^; of the attempts by local providers to make better use of the different forms of evidence in the planning of public services^4^ and on the multiple processes, tasks and people involved in incorporating research into delivering healthcare services.^5^, ^6^ This disconnect between research and practice can be conceptualised broadly as^7^: a knowledge transfer problem, where knowledge is ‘pushed’ from researchers or ‘pulled’ by actors within organisations, or a knowledge production problem, where academic and organisational ways of knowing are brought together to create new knowledge.

### 1.2 Knowledge translation, mobilisation and brokering

The positioning of knowledge in use and in practice appears on a continuum: on one side, a simple transaction between researchers and practitioners; on the other, end‐users are fully involved in bringing together different types of knowledge, facilitated to shape that knowledge.^5^, ^8^ Knowledge brokering is a combination of activities: managing information such as research evidence and data; linking and exchanging ideas between the different knowledge stakeholders and capacity building to utilise research evidence to enact positive change.^9^ Knowledge mobilisation has often relied on brokering to facilitate ‘bridging the gap’ between researchers and practitioners or policymakers, although arguably a reliance on designated brokers creates a process dependent on their specific skills, networks and preferences.^8^, ^9^ Alternatively, knowledge brokering is a collective process, enacted within the team, enabled inside organisations and strengthened by the inclusion of a broad range of research users coproducing and mobilising evidence relevant to their local environments.^9^

### 1.3 The role of coproduction in mobilising knowledge

Coproduction has been developed and employed in policy and practice across many disciplines, including environment, sustainability and health, motivated by the need to address complex problems, where the knowledge required to generate solutions requires collaboration between researchers and nonacademic end‐users of the research.^10^, ^11^ While there is some agreement across the disciplines that coproduction embraces a range of practices across different levels of engagement, descriptions and definitions vary.^11^, ^12^, ^13^, ^14^ Smith et al.^15^ present a typology of coproduction to explain the different ways that coproduction is defined and employed: citizens' contributions to public services, where public services are reliant on voluntary contributions for success; integrated knowledge translation, where academic researchers work with end users with the aim of making research more valid; equitable and experientially informed research, where experiential knowledge is seen as crucial to the research process. Coproduction demands that the knowledge and experience that end‐users bring is valued on a par with that of the traditional knowledge producers; that relationships are reciprocal and mutually beneficial, achieving more together than would be possible apart, and to be facilitated to do so by networks, organisations and a resource infrastructure that enables involvement.^8^, ^11^

There is general agreement that coproduction adds value through shaping how knowledge is generated, understood and utilised in the design and delivery of impactful public services; bringing together the multiple perspectives and skills of stakeholders and addressing the imbalances in power by respecting and valuing the knowledge they bring to discussions; providing contributors with the ‘space to talk’ and ‘space to change’.^8^, ^16^, ^17^, ^18^ Nonetheless, tensions arise where the vision of coproduction and the reality of local context intersect and consequently, coproduction is not free of risk or cost.^10^, ^19^ Recent reviews of the use of coproduction in health research suggest that coproduction can benefit the research process although little evidence exists to show it has improved the management of health conditions.^12^, ^20^ Whether or not there is a need for robust evaluation to identify whether coproduction produces improved health outcomes is a point of debate. Williams et al.^21^ argue from a democratic stance that the normative desirability of coproduction does not demand a sound evidence base, while a technocratic position would require empirical evidence to substantiate the benefits of the approach.

### 1.4 The context of our study

As a partnership between universities and healthcare organisations (including NHS and local government), The National Institute for Health Research (NIHR) Collaboration for Leadership in Applied Health Research and Care North West Coast (CLAHRC‐NWC) focused on improving patient outcomes through the conduct and implementation of applied health research. Increasing the capacity of partners to undertake and act on the findings of applied health research is a core function of the 13 CLAHRCs situated across England. A particular focus of CLAHRC‐NWC was reducing health inequalities, since the region faces some of the starkest variations across England, with average life expectancy differing by up to 12 years. Although widely acknowledged that social injustices and resulting health inequalities are unnecessary, there is a lack of common ground on definitions, which hinders effective action.^22^ A focus on reducing inequalities is essential in advancing population health furthermore integrating an equity focus into projects is necessary to spend public money ‘wisely’.^23^ However, relatively little evidence has an explicit focus on equity, and some policies and interventions may inadvertently differentially benefit more socioeconomically advantaged groups.^23^

With its NHS and Local Authority partners, CLAHRC‐NWC coproduced a programme focussing on a shared strategic priority to identify and evaluate local healthcare treatments and services aiming to reduce emergency admissions. The goal of this Partners Priority Programme (PPP) was to support teams to develop the capacity to evaluate the delivery of their own services enabling organisations to assemble timely, practical and relevant evidence that could feed directly into local decision‐making. By encouraging mixed teams to work together to coproduce their evaluation, the PPP also aimed to systematise the consideration of health inequalities in service change.^24^

### 1.5 Description of the facilitated interactive group learning approach

The PPP was achieved through a series of eight, 1‐day workshops over a 12‐month period, on evaluation that brought academics, professionals and users of services together in Collaborative Implementation Groups (CIGs), with an emphasis on interactive and collaborative co/peer learning.^24^ Teams from a variety of organisations providing or commissioning healthcare brought the projects that they wished to evaluate, were assigned to a thematic CIG and their support and learning were facilitated by a dedicated member of the academic team.

Workshop sessions provided teams with structured but flexible academic support to coproduce their equity‐sensitive evaluations. The first four workshops covered the topics needed to support each project team to develop an equity‐sensitive evaluation plan, with subsequent workshops including sessions to support: operationalising evaluation plans; communication and actioning of their findings; dissemination to a wider audience.

An evaluation workbook, incorporating the Health Inequalities Assessment Toolkit (www.HIAT.org.uk), was compiled and provided to all participants of the workshops as a resource; initially provided incrementally, following feedback from the first cohort, the workbook was provided at the start of the workshop series for cohort two.

Two cohorts took part: 9 teams were supported during 2016–2017 and 16 during 2017–2018. Participants (101 across 42 organisations) conducted evaluations of projects planned or ongoing in their local health system. Projects covered a wide range of services (e.g., enhanced primary care, social prescribing, clinicians in the community), conditions (e.g., cancer, dementia, diabetes, motor neurone disease) and settings. In addition to the Evaluation Workshops, 23 interns were supported with part salary‐backfill and additional training in research methods to carry out a small research project aligned with the evaluation.

Coproduced outputs include 14 internal evaluation reports and 10 peer‐reviewed articles: A supplementary table lists the projects, the types of team members and the outputs they completed. Not all project teams were able to produce an evaluation report for a variety of reasons including early withdrawal of the particular service, changes in project team personnel and staff sickness. However, those reports and journal articles that were produced did respond to health inequalities.

### 1.6 Aim of the study

Our research study seeks to contribute to the evidence base on practising coproduction for knowledge mobilisation. Specifically, we explored our facilitated interactive group learning approach to enhancing the equity‐sensitive evaluation of local healthcare services:

1. What was the experience of participants of the PPP and the CIGs?
2. How was knowledge mobilisation achieved? What, why and by whom?
3. What are the key elements that enhance the process of coproducing equity‐sensitive evaluations?

We used as our ‘lens’ a framework for knowledge mobilisers^25^ that was derived from a review of the diverse and fragmented literature on models and tools, to develop a clear description of the role of the PPP/CIG approach.

## 2 METHODS

We conducted an exploratory study with the participants, facilitators and ‘design team’ of two cohorts of the programme.

### 2.1 Data collection

We invited all PPP participants to take part, including public advisors (PAs), interns and facilitators along with organisational leads (R&D and line managers) to share their views. We collected data from each cohort separately using focus groups (FG) and semistructured interviews (S‐SI). FGs were held for PAs, interns, project team leads and academic facilitators. All of the FGs included representation of participants from different projects across the programme (Table 1).

**Table 1:** Participants who took part within each focus group (from Saini et al.^24^).

Participants	Cohort 1 (Nov 2016 to Oct 2017), *n* (%)	Cohort 2 (Jul 2017 to Jun 2018), *n* (%)
Public advisors	5/16 (31)	4/27 (15)
Interns	5/11 (45)	4/14 (29)
Partner leads	6/11(55)	0/14 (0)
Facilitators	8/8 (100)	8/12 (67)
Research and development managers	NA^a^	4/4 (100)
PPP design team members	NA^a^	6/6 (100)

Abbreviation: NA, not applicable; PPP, Partners Priority Programme.

^a^ Partner leads, research and development staff and the design team members were interviewed about both cohorts 1 and 2.

We conducted FGs following the final workshop sessions; participants attended the group they felt was most relevant to them, with each lasting for 1 h, and where they were asked to talk about their experiences of the programme. The study was conducted in English, and interviews and FGs were recorded using a digital audio recorder and transcribed verbatim.

Six months after the final workshop we scheduled S‐SI with representatives (in most cases the lead) of each team participating in the first cohort: each interviewee was asked to discuss the impacts of the programme on their project, team and organisation.

### 2.2 Data analysis and interpretation

We used a reflexive thematic analysis approach^26^ to identify themes and patterns across our data, recognising the value of this method's flexibility and potential to enable a rich and detailed account to be shaped. Following transcription, all authors read the transcripts, making notes of interesting features in the data and reflecting on potential codes with particular attention being paid to the four questions posed by the framework for knowledge mobilisers (Table 2). In using this framework for (deductive) initial coding, we were able to connect the data with a range of viewpoints of knowledge mobilisation. Subsequent analysis, refinement of codes and generation of themes were inductive, that is, informed by but independent of Ward's framework. Transcripts were imported into NVivo11/12 for ease of coding; codes were collated, developed and refined throughout the analysis period. All authors contributed to generating the final themes.

**Table 2:** A framework for knowledge mobilisers.^25^

1. Why is knowledge being mobilised?	To develop local solutions to practice‐based problems; to develop new policies, programmes and/or recommendations; to adopt/implement clearly defined practices and policies; to change practices and behaviours; to produce useful research/scientific knowledge)
2. Whose knowledge is being mobilised?	Professional knowledge producers who produce empirical and/or theoretical knowledge and evidence; frontline practitioners and service providers responsible for delivering services to members of the public; members of the public acting as or on behalf of their communities and people in receipt of services; decision makers responsible for commissioning services and/or designing local/regional/national policies and strategies; product and programme developers responsible for designing, producing and/or implementing tangible products, services and programmes
3. What type of knowledge is being mobilised?	Scientific/factual knowledge—research findings, quality and performance data, population data and statistics, evaluation data; technical knowledge—practical skills, experiences and expertise; practical wisdom—professional judgments, values beliefs
4. How is knowledge being mobilised?	Making connections between knowledge stakeholders and actors by establishing and brokering relationships Disseminating and synthesising knowledge via online databases, communication strategies and evidence synthesis services Facilitating interactive learning and coproduction via participatory research projects and action learning sets

## 3 FINDINGS

Through the initial mapping of our findings against Ward's categories, we were able to characterise the ‘why, whose, what and how’ of knowledge mobilisation during (and subsequent to) the workshop programme. The motivations behind the programme of workshops were to ‘develop local solutions’ to reducing emergency admissions to hospital care and also to ‘change practices and behaviour’ to employ a robust evaluation to support equity‐sensitive decisions on transforming services. The knowledge being mobilised came from those that are responsible for delivering services, members of the public on behalf of those in receipt of services, those responsible for commissioning services as well as from academic/professional knowledge producers. We also saw examples of ‘technical knowledge’ and ‘practical wisdom’ being brought to the CIGs, contributing to the production of ‘scientific/factual knowledge’ in the form of internal evaluation reports and journal articles. It was also clear from the transcripts that the means by which knowledge was mobilised, was through ‘facilitating interactive learning and coproduction’ and by ‘brokering relationships’ between those with relevant data, practical skills, experiences and expertise.

Following initial coding, we developed four themes to illustrate the activities and processes taking place: (1) Creating the setting for coproduction and knowledge mobilisation; (2) establishing a common purpose, meaning and language for reducing health inequalities; (3) making connections and brokering relationships and (4) challenging and transforming the role of evaluation.

### 3.1 Theme 1: Creating a setting for coproduction and knowledge mobilisation

The venue of the evaluation workshops established the space or environment for negotiating coproduction, bringing the various stakeholders together, out of their day‐to‐day setting, and into CIGs to exchange knowledge. What brought meaning to the training was a focus on the participants' own projects, while acknowledging that placing teams with similar projects into the same CIG was beneficial for information sharing and mutual learning;

it's partly the time out from your everyday business where if you tried to do this you'd just get sucked up in to everything else that's your priorities it's being actually physically out of work and sat within the environment specifically to work on the evaluation and the process itself that I think is really valuable (FG Project Lead Cohort 1)

the CCG1 on the same table as the CCG2 and that worked really well because city2 were trying to do what we had done here in city1 … 5 years earlier (FG R&D Group Cohort 1)

However, the CIGs did not always progress to the wider collaborative support that was envisioned, for example, if membership of the group was unstable, or where interactive activities felt contrived.

we've never really been a support network we don't really know who is in our CIG except for when we turn up to a workshop and there's someone sat at our table and sometimes its different people sometimes there's just us (FG Project Lead Cohort 1)

the CIGs, I think it's a good idea, but it just doesn't always seem to work out that way and trying to force it. It doesn't seem very natural sometimes to have those conversations (FG R&D Group Cohort 1)

### 3.2 Theme 2: Challenging and transforming the role of evaluation in improving and commissioning services

Study participants acknowledged their motivation was to highlight the effectiveness of their services and in turn influence the people with the ‘power’ to adopt or continue to commission. Participants indicated that taking part gave them the opportunity to widen the scope and rigour of their evaluation, increasing the relevance and value to local stakeholders and also confidence in the decisions to change or maintain, aspects of their service.

our initiative has been running for six years now so each year we've looked at the numbers the quantitative side but never really done those in‐depth interviews with stakeholders and they've really welcomed that opportunity I think to have their say …. how they feel, the impact that it's made (FG Project Lead Cohort 1)

The evaluation actually meant that we didn't make the service change. We saw the value in what we were offering and [that] we weren't duplicating. The service users and carers really valued what we were doing. There was a good rationale for it, so we didn't actually make the service change that we had [originally] considered (S‐SI 1)

By including a wider range of knowledge stakeholders in their evaluations, some participants realised that improvements in the way that their organisation routinely collects and shares information are needed to strengthen their evidence base for service change.

its…making sure that we carry on evaluating what we do and trying to improve what we do …any evidence we produce get it out there and make sure that we get it to the right people I think to improve service (FG Interns Cohort 1)

Participating in the workshops raised their awareness of the different types of knowledge available and how these could be accessed. Participants reflected that the PPP/CIG had helped to unlock available ‘scientific/factual’ knowledge (e.g., research findings; quality and performance data) and that there is purpose in marrying this with their own ‘technical’ knowledge thus producing evidence recognised by all stakeholders.

it's opened me up to research more than even more than I ever thought I would …. there's things out there I would never have read, there's databases out there I would never have gone and looked on, there's things I've used that I would never have thought of (FG Interns Cohort 1)

gathered evidence in a way that was understandable to clinicians and …. presented in a way the organisation understood (S‐SI 2)

Being part of the PPP granted participants ‘permission’ and the skills to bring in different types and sources of evidence to their evaluations, including using methods that are often seen as resource intensive or challenging.

no one has ever, in my opinion, in this organisation appreciated qualitative research before, ever because, we said in the presentation we did for the team, if you'd have said I'm going to go out and have a chat to a practice and see how they feel about this. There's just so little time in the day people (S‐SI 6)

On completing the programme, it was evident that participants recognised that improvements in the process of commissioning services were also needed and that implementing ‘top down’ change without a good evidence base or evaluation plan is an opportunity cost.

the trouble is its cultural isn't it … I think of several projects that we are having to do now, mandated, where there is no evidence that they're going to work but we've got to do them which is taking away time from evaluation of other things that might be worthwhile so it's all a national problem (FG Project Lead Cohort 1)

I think it just reinforces the value of evaluating things properly, I think in the NHS we make changes and then we decide that they've worked … without really doing the necessary evaluation to really find that out. We were able to disseminate that … it's really worth taking the time to do that evaluation properly because otherwise you're making service changes without really understanding what you're doing (S‐SI 1)

### 3.3 Theme 3: Establishing a common purpose, meaning and language for reducing health inequalities

The support given to embed a focus on health inequalities into their evaluation would also help participants to pilot and deliver services that reflected everyone's needs: patients, communities and healthcare organisations. Some participants indicated that the support had helped to re‐energise commitments towards reducing health inequalities.

we probably did let slip inequalities and I think having that particular session has made us think we do really want to include that and we need to go back and figure out how we can include that in our model in a meaningful way that we can report on and hopefully see a difference (FG Project Lead Cohort 1)

we've … changed our thinking … we're now thinking about being more proactive in reporting outcomes in such a way as people can see the health inequalities related to them rather than just reporting the overall number (S‐SI 6)

Being part of the programme encouraged deeper thinking about action on health inequalities and also raised the potential to incorporate people's experiences of their health conditions into improving practice and services. Participating in the programme gave evaluation teams the tools and the confidence to clarify how they would identify and evaluate health inequalities.

we used the HIAT … it really facilitated a big discussion around health inequalities and how members of the multidisciplinary team are thinking about it and then applying it to their practice now. And in so just by raising it, I can't say that its embedded in their practice, but they certainly are taking it on board (FG Intern Cohort 2)

### 3.4 Theme 4: Making connections and brokering relationships: Mobilising knowledge from multiple contributors

Within the data is a broad description of exchange and mobilisation between different groups of knowledge stakeholders—academics, frontline practitioners, service providers and members of the public—reflecting the multiple outcomes to be achieved by the PPP and the individual teams. The CIGs and their formal meetings were arranged to facilitate the forging of new links not only within the groups but also identifying more widely individuals to collaborate and work with across the programme.

each project, each local team would come and be working on evaluating their initiative but that we used that as the kind of vehicle for … cross learning, so … you'd say Project01 needs to go and talk to … and you'd start to build those links from the bottom up (FG Design Team Cohort 1)

Participants reflected on the potential for reciprocal relationships between practice and academia, the value of bringing together their research and technical knowledge to influence practice and sustaining those connections to help solve problems in the future.

so many people from an academic point of view that want to produce the research and so many people from an NHS point of view that have got the opportunity that have got the data and the people and the knowledge of it so putting the two together actually makes perfect sense (FG Interns Cohort 1)

we've established some great links I know our researcher now (facilitator name), is really supportive and knowledgeable and helping with all sort of things outside of this and also the public advisor (FG Interns Cohort 1).

The CIG approach placed great emphasis on teams recruiting members of the public to be involved in their evaluation, in some cases leading to particular aspects of the project. On the whole, involving members of the public, their personal experiences and practical skills was viewed as a positive experience.

so they are going to carry out the project themselves so an expert by experience, they've got experience of being admitted to [a] ward and now they are going to ask service users about their perceptions and experience (FG R&D Group Cohort 1)

he was absolutely excellent because he lives within the locality where the initiatives were offered so he had information …. that I probably otherwise wouldn't have had ready access to … how much bus services cost, how much taxis cost, really, really useful (S‐SI 1)

Participants also reflected on the utility of including stakeholders from the beginning in decisions on what knowledge could be gathered relevant to the evaluation, but also more generally. Involving PAs in the programme and CIGs enabled their practical knowledge to be built‐in to the data collection and dissemination activities, as well as directly informing the findings, with the patient ‘voice’ enriching how professionals/practitioners consider and articulate health and wellbeing.

it's often the case we get involved at the very end … ‘now pull me the numbers for that’ but we wasn't involved in the outcomes, but could have told you that's not measurable (FG Project Lead Cohort 1)

it's got me passionate about … listening to the patients and wanting the patient's journey to be different ultimately and more beneficial for all of us (FG Interns Cohort 1)

Barriers to mobilising knowledge from multiple stakeholders were also acknowledged. Frontline practitioners, for example, are often required to prioritise responding to clinical demands ahead of research and evaluation.

people have been hopefully given the freedom to come out and do these projects but equally you know there are clinical pressures in every setting … and when they have come back, well you know that was great well done you know get back to the clinical job we haven't got time for research (FG R&D Group Cohort 1)

Late engagement of PAs and topic experts, when evaluation plans were already well‐developed meant that their contributions could have been less tokenistic and more wide‐ranging, emphasising the need for those with relevant factual, technical or practical knowledge to be included early on in the mobilisation process to realise the full benefits.

it was already shaped before we started PPP so we've had to, not necessarily remould it but we've had to rejig it in order to accommodate the public advisor which was great but it also feels like that could have been really sort of addressed earlier on in the process (FG Project lead cohort 1)

we ran into lots of issues with [….] trying to advise us to alter what we'd planned to do and whether we needed [….] approval. I found all of that quite stressful and I think if that relationship had been established much earlier on it could all have been done as part of one process (FG Interns Cohort 1)

In some instances, bringing in the ‘voice’ of patients and the public was challenging, both ethically and practically with some expressing frustration with teams' apparent inability or unwillingness to utilise their skills, for example in handling NHS data.

they said well you can do anything but as it turned out anything was in inverted commas because when it came to for example … data analysis, … they said oh no you can't be involved in that because it's sensitive data (FG2 Public Advisor Cohort 1)

Particular challenges with involving vulnerable patient groups were also apparent, although we saw that teams adopted a flexible approach by, for example, involving carers in their project.

there needs to be an awareness that it is not always easy to find somebody who can … take on the sort of formal role that the [programme] is expecting because we are looking at the area of [neurological condition] and the challenges that presents in finding somebody (FG R&D Group Cohort 1)

## 4 DISCUSSION

Delivery of the PPP evaluation workshops through CIGs supported multi‐faceted teams to evaluate local solutions to reducing emergency admissions to hospital care and enhance practices of equity‐sensitive transformation of services. By facilitating interactive learning, coproduction processes and relationships, knowledge was mobilised between those with relevant data, practical skills, experiences and expertise. Technical and experiential knowledge were brought together and contributed to the production of ‘scientific’ knowledge in evaluation reports and journal articles. Coproduction took place between those responsible for delivering services, members of the public, commissioners and academic researchers. The CIG approach has many of the attributes of ‘equitable and experientially‐informed research’ positioning people with relevant experiential knowledge as essential partners in the coproduction process.^15^

We identified four themes to illustrate how the CIG approach to delivering intensive and facilitated training supported equity‐sensitive evaluations of local healthcare services that informed local decision‐making by (1) creating the setting; (2) establishing a common purpose; (3) making connections and (4) challenging and transforming the role of evaluation.

Our study demonstrates a practical example of engaged scholarship, offering resources, training and assistance with methodological issues that commissioners and practitioners find valuable in their local evaluations, thereby developing a relationship of reciprocity and mutual benefit.^6^ This ‘middle‐ground research’, involving close collaboration between academics, policymakers, managers/frontline staff, and patients, aims to shorten the time taken for healthcare organisations to implement research findings.^2^ The programme gave participants the tools and confidence to address their organisation's aims of reducing health inequalities, and by mobilising the full range of knowledge available to them to support their local evaluations. Furthermore, involving patients and carers at the earliest opportunity acknowledges that addressing health inequalities requires understanding the perspective of those experiencing social and health inequalities.^23^

CIGs delineated a space to negotiate coproduction and focus on their own evaluations alongside peers with a similar focus and interest. Facilitators were able to advocate for the different perspectives that a wide range of stakeholders bring to the value and relevance of the process of evaluation but also in interpreting and implementing their findings. Consistent with the findings of Clarke et al.,^27^ where formal and funded facilitators help maintain the coproduction process, our facilitated CIGs were largely successful in supporting teams to coproduce evaluations of their services. For individual researchers, the ability to embrace more equal power‐sharing, accept different ways of locating evidence, negotiate and communicate effectively and manage relationships with stakeholders are key skills and competencies to enhance coproduction.^28^

Being ‘equal partners’ is an important foundation for effective coproduction, and early involvement of service users in the planning would have been an opportunity to discuss alternative ways of evaluating projects.^10^, ^17^ Furthermore, involving patients and carers at the earliest opportunity acknowledges that addressing health inequalities entails understanding the perspective of those with lived experience of inequality.^23^ This was reflected in our study, where engagement of PAs into the team once the evaluation plan was developed, rather than being core members from the start, negatively influenced how they perceived their contribution. Similarly, we observed constraints on public involvement in evaluation that reflected barriers linked to the governance, accountability and the hierarchical nature of applied health research, such as data confidentiality.

Healthcare professionals will typically work in interdisciplinary teams, where a shared language and understanding are key to providing good care, offering a compelling argument for mixed‐team professional development.^29^ As others^30^ have suggested, a collaborative approach encourages learners to work together to search for understanding by examining problems and discussing solutions. In CIGs, ‘learners’ worked in small groups to discuss the material in the evaluation workbook and its application to their own projects. Involving PAs in the CIGs brought a more diverse perspective to the evaluations, and it was clear that the professionals valued the knowledge that was shared; this contributes further to the evidence base that patients and professionals learning together can support innovation and improvement in health and social care.

The PPP and the CIG approach can be considered a potential ‘game changer’, where commissioners/clinicians find new and more productive ways of implementing research through working in collaboration with patients/carers and researchers.^14^ Engaging individuals and organisations in collaborative processes requires a clear vision of ‘what's in it for them’, with incentives for a researcher (e.g., the needs of their academic career path),^10^ being different from those of a professional (e.g., making a difference in their practice) or a patient (e.g., improving the experience of service‐users).^8^ In common with others,^29^ we found that providing the space and time away from a highly pressured workplace and linking the content of the training to issues of importance to them enabled clinicians/professionals to focus and learn effectively and in some cases sustain the connections made beyond the formal programme. However, we observed that the consistent engagement of busy clinicians was a challenge, in line with others^3^ who highlighted that health and social care professional are frequently faced with a lack of institutional support to participate in research, including competing demands on their time, as well as dwindling resources.

CIGs supported elements of the principles of coproduction^14^, ^28^: participants reflected on the reciprocity and mutual benefit that more active involvement of patients and carers had on the relationships they formed; the evaluations were primarily driven by the needs of the end‐users; that developing capacity for patients to contribute to the evaluation and facilitating organisations to support their active involvement would, in turn, strengthen and advocate the transformation of services; diverse perspectives and knowledge were represented in academic outputs (publications). In this way, CIGs may provide a space where ‘expert’ and ‘lay’ can interact on an equal footing, enhancing the mobilisation of experiential knowledge. Coproduction was seen as authentic since participants could appreciate the difference that embedding a focus on health inequalities and the views of service users into their evaluation reports had made, that is, the embodiment of coproduction into an ‘actionable output’.^16^ By bringing together multifaceted teams, CIGs facilitated collective knowledge brokering^9^ whereby evidence‐informed transformation of local healthcare services is enhanced by the coproduction of a robust evaluation.

While the challenge of health equity and the social determinants of health can feel overwhelming where there isn't a shared understanding of the problem,^31^ our findings reflect those of Waring et al.^32^ who suggest that by establishing a common language (syntactic brokering) through, for example, the use of HIAT, common meaning may be established (semantic brokering), which in turn could provide a foundation for establishing common purpose (pragmatic brokering) of integrating an equity focus into evaluation. The PPP/CIG approach, which incorporated facilitated learning on equity‐sensitive methods for evaluation, would answer the call from Sabey et al.^29^ to further engage with the wider determinants of health and commissioning in the primary and community sectors. As McMahon^31^ advocates, the positive experience of collaboration within the PPP and sharing the history of that success as a multiorganisational partnership is essential in enabling collective action on health inequalities.

### 4.1 Strengths

This study has been coproduced with our partners from the conception of the PPP, throughout the research of the workshops, and on to the development of the findings and writing of this article.

We have used established framings of coproduction and knowledge mobilisation to understand how the PPP, through CIGs, facilitated the engagement of academics, commissioners/practitioners and members of the public to support the design, evaluation and implementation of healthcare services. The workshop programme was delivered over a period of 12 months to teams evaluating projects that encompassed a wide range of settings (e.g. neonatal care, clinicians in the community, rehabilitation) and conditions (e.g., dementia, motor neurone disease and cardio vascular disease). Participants comprised 23 teams from a range of organisations (NHS, local authorities, third sector). To our knowledge, this is the first example of an in‐depth, practical programme to support a diverse audience to evaluate the transformation of local healthcare services. Our findings are, therefore, likely to be widely applicable across a range of health and care settings and topics, being particularly suitable for service redesign in a challenging financial environment brought about through austerity and exacerbated by the pandemic.

Our analysis utilised Ward's framework^25^ for knowledge mobilisers to clarify and underpin, taking the questions and accompanying categories to guide our interpretation of what participants said about their experiences of the programme. The framework has been valuable in developing a greater awareness of the circumstances that led to the successful coproduction of knowledge to support equity‐sensitive evaluations: establishing a common purpose and location where the full range (scientific, technical, experiential) of knowledge donors are brought together and given the tools and confidence to plan an evaluation that addresses health inequalities.

### 4.2 Limitations

As acknowledged by Saini et al.,^24^ we cannot rule out the possibility that those taking part in the interviews, FGs and survey were not representative of all that took part in the workshops. Our study concentrated on participants’ self‐reported experiences of the programme through interviews and FGs: observations of the workshop sessions may have improved the independence of the data and findings.

## 5 CONCLUSIONS

Increasingly, healthcare systems are mandated to play a more central role in addressing health inequalities recognising the financial cost of, and the rising demand for, services due to preventable ill health.^31^ We report on a practical example of engaged scholarship, where teams were supported with resources, interactive training and methodological advice to evaluate their own services enabling organisations to assemble timely practical and relevant evidence that could feed directly into local decision‐making. By encouraging mixed teams of practitioners, commissioners, patients, the public and researchers to coproduce evaluation plans, the PPP also aimed to systematise health equity into service change. The findings of our study illustrate that the CIG approach to delivering training gave participants the tools and confidence to address their organisation's stated aims of reducing health inequalities, by mobilising knowledge from a range of stakeholders to coproduce evaluations of their local services. As NIHR comes to focus more on community and social care research, this will likely require more participatory approaches to research and evaluation where public partners can more readily play an active role, build quality relationships and feel supported and confident to share their knowledge,^15^ which we have shown the CIG approach can facilitate. While our study sought to determine the short‐term effects of the workshop programme, further research should consider the wider impact on participants, their organisations and the principles of coproduction to refine our understanding of how collaborative learning in general and CIGs in particular, can contribute to innovation in health and social care. Workshops were delivered face‐to‐face, and so acknowledging that working practices have changed as a result of the covid‐19 pandemic, research should investigate whether the CIG approach is amenable to delivery online.

## AUTHOR CONTRIBUTIONS

Mark Gabbay led the research study. Interviews and focus groups were conducted by Shaima Hassan and Pooja Saini. Transcripts were coded by Jane Cloke, Shaima Hassan, Mark Gabbay, Mark Goodall and Adele Ring. All authors (Jane Cloke, Shaima Hassan, Mark Gabbay, Mark Goodall, Adele Ring, Naheed Tahir, Pooja Saini) contributed to research team discussions, developing the topic guide, and contributed to further analysis of the data to develop themes. Jane Cloke, Shaima Hassan, and MBG wrote the manuscript. All authors read through drafts of the manuscript and approved the final version.

## CONFLICT OF INTEREST STATEMENT

The authors declare no conflict of interest.

## ETHICS STATEMENT

Ethical approval was obtained from the University of Liverpool Ethics Research Committee (Reference Number: 2236). All participants were informed about the study via an invitation email that provided details of the study involving focus group discussions and an online survey a participant information sheet, and a consent form. All participants provided informed consent and the study was conducted in English.

## Supporting information

Supporting information.

## Data Availability

The datasets generated and/or analysed during the current study are not publicly available due to ethical restrictions but are available from the corresponding author upon reasonable request.
