# Supplementary material for: Tapping into the power of coproduction and knowledge mobilisation: Exploration of a facilitated interactive group learning approach to support equity‐sensitive decision‐making in local health and care services
Source: Health Expect. 2023 May 8;26(4):1692–702. doi: 10.1111/hex.13774 (PMC10349235; doi:10.1111/hex.13774)
Supplement: Supplementary file 1 — Supporting information. [file HEX-26--s001.docx]

**Supplementary material: Outputs from Cohorts one and two of the Partners Priority Programme**

| **Project** | **PPP Cohort** | **Composition of the project team** | | | **Outputs** |
| --- | --- | --- | --- | --- | --- |
|  |  | **Number of Professionals** | **Number of researchers** | **Number of service users and carers** |  |
| Evaluation of the impacts of the Knowsley CVD service, Liverpool Heart and Chest Hospital. | 1 | 3 | 1 | 2 | 1) Internal evaluation report  2) Impact of a community-based cardiovascular disease service intervention in a highly deprived area(1) |
| Evaluation of the impacts of the Knowsley COPD service, Liverpool Heart and Chest Hospital. | 1 | 3 | 1 | 1 | 1) Internal evaluation report  2) Impact of community-based chronic obstructive pulmonary disease service, a multidisciplinary intervention in an area of high deprivation: a longitudinal matched controlled study(2) |
| To explore the impacts of a system-wide diabetes care partnership, Liverpool Diabetes Partnership. | 1 | 3 | 1 | 1 | Internal evaluation report |
| Evaluation of Liverpool GP specification (a quality contract) upon the key areas of healthcare activity, quality of general practice, and patient experience over a 10-year period | 1 | 4 | 2 | 2 | 1) Internal evaluation report  2) Evaluating the Effectiveness of a Local Primary Care Incentive Scheme: A Difference-in-Differences Study(3) |
| Evaluation of the impacts of multi-disciplinary integrated Community Care Teams. | 1 | 2 | 2 | 1 | Internal evaluation report |
| Preventing emergency admissions among cancer patients | 1 | 2 | 1 | 1 | Internal evaluation report |
| Evaluation of clinical decision-making in the use of inpatient mental health beds, Cheshire Wirral Partnership NHS Trust | 1 | 4 | 1 | 1 | 1) Internal evaluation report  2) Use of Acute Psychiatric Hospitalisation: A Study of the Factors Influencing Decisions to Arrange Acute Admission to Inpatient Mental Health Facilities(4) |
| Evaluation of the impacts of the Life Rooms on the recovery of Mersey Care service users, Liverpool Mersey Care. | 2 | 1 | 2 | 1 | 1) Internal evaluation report  2) Social prescribing for people with mental health needs living in disadvantaged communities: the Life Rooms model(5) |
| Evaluation of Wigan Later Life and Memory Service (LLAMS) – improving young onset dementia (YOD) services, North West Boroughs NHS Trust | 2 | 1 | 2 | 2 | 1) Internal evaluation report  2) Evaluating a young-onset dementia service from two sides of the coin: staff and service user perspectives(6) |
| Overarching evaluation framework for public health mental health and wellbeing interventions, Public Health England. | 2 | 5 | 2 | 1 | No final report |
| Evaluation of a program to enhance wellbeing and quality of Life in Motor Neurone Disease (MND) patients, The Walton Centre, Liverpool | 2 | 2 | 2 | 1 | 1) Internal evaluation report  2) Communicating psychosocial well-being in motor neurone disease to staff: results from a World Café approach(7) |
| Evaluation of the STEP (Succeed, Thrive, Empower Pennine) Service, Blackburn with Darwin CCG and Blackburn with Darwin Council. | 2 | 5 | 2 | 2 | No final report |
| Evaluation of the use of home phototherapy as a treatment for physiological jaundice, Liverpool Women’s Hospital | 2 | 2 | 2 | 1 | No final report |
| Evaluating of advice on prescription (social prescribing service): providing income maximisation advice in primary care settings, Liverpool CCG | 2 | 3 | 1 | 2 | No final report |
| Evaluation of a programme for early supported discharge of well, late preterm babies, Lancashire Teaching Hospitals NHS Trust | 2 | 9 | 1 | 2 | Internal evaluation report |
| To explore service users’ experiences of the personality disorders hub and its impact on their overall mental health and wellbeing, Liverpool Mersey Care | 2 | 3 | 1 | 4 | Internal evaluation report  A preliminary service evaluation of a personality disorder case management service(8) |
| Evaluation of the Youth Information and Counselling (YIAC) Model, Liverpool CCG). | 2 | 2 | 1 | 3 | 1) Internal evaluation report  2) An exploration of young people’s, parent/carers’, and professionals’ experiences of a voluntary sector organisation operating a Youth Information, Advice, and Counselling (YIAC) model in a disadvantaged area(9) |
| Evaluation of Sefton public sector reform programme; early intervention & prevention (EIP), and community connector project, Sefton Council, Liverpool. | 2 | 2 | 2 | 2 | 1) Internal evaluation report  2) Enabling middle-aged and older adults accessing community services to reduce social isolation: Community Connectors(10) |
| Identification of factors that contribute to emergency re-admissions to hospital for older patients having received inpatient rehabilitative care (Better Care Now, Blackpool Teaching Hospital) | 1 and 2 | 2 | 1 | 2 | Internal evaluation report |
| Delivery of high quality primary care at scale and improving access in Blackburn and Darwin | 1 | 2 | 1 | 1 | No final report |
| Evaluation of the impacts and effectiveness of each Multidisciplinary Team (MDT), Community Health Services, East Lancashire. | 1 | 1 | 1 | 3 | No final report |
| Evaluation of a community integrated mental health and physical health service, 5 Boroughs NHS Partnership Mental Health Services. | 1 | 2 | 1 | 1 | Internal evaluation report |
| Evaluation of Telehealth for COPD : Re-design of respiratory services in Liverpool, Liverpool CCG | 2 | 2 | 1 | 2 | No final report |
| Evaluation of Multidisciplinary Team (MDT) working in Integrated Care, Liverpool Heart and Chest Hospital | 2 | 1 | 2 | 3 | No final report |
| To gain an insight into the experience of service users admitted to an inpatient psychiatric ward, Cheshire Wirral Partnership NHS Trust. | 2 | 3 | 1 | 4 | Internal evaluation report |

References

1. Downing J, Rose TC, Saini P, Matata B, McIntosh Z, Comerford T, et al. Impact of a community-based cardiovascular disease service intervention in a highly deprived area. Heart. 2020;106(5):374.

2. Saini P, Rose T, Downing J, Matata B, Pilsworth S, Pemberton A, et al. Impact of community-based chronic obstructive pulmonary disease service, a multidisciplinary intervention in an area of high deprivation: a longitudinal matched controlled study. BMJ Open. 2020;10(5):e032931.

3. Khedmati Morasae E, Rose TC, Gabbay M, Buckels L, Morris C, Poll S, et al. Evaluating the Effectiveness of a Local Primary Care Incentive Scheme: A Difference-in-Differences Study. Medical Care Research and Review. 2022;79(3):394-403.

4. Nathan R, Gabbay M, Boyle S, Elliott P, Giebel C, O'Loughlin C, et al. Use of Acute Psychiatric Hospitalisation: A Study of the Factors Influencing Decisions to Arrange Acute Admission to Inpatient Mental Health Facilities. Front Psychiatry. 2021;12:696478-.

5. Hassan SM, Giebel C, Morasae EK, Rotheram C, Mathieson V, Ward D, et al. Social prescribing for people with mental health needs living in disadvantaged communities: the Life Rooms model. BMC Health Services Research. 2020;20(1):19.

6. Giebel C, Eastham C, Cannon J, Wilson J, Wilson J, Pearson A. Evaluating a young-onset dementia service from two sides of the coin: staff and service user perspectives. BMC Health Services Research. 2020;20(1):187.

7. Giebel C, Medley G, Smith S, Thornton M, Furlong M, Ennis M, et al. Communicating psychosocial well-being in motor neurone disease to staff: results from a World Café approach. Quality of Life Research. 2019;28(9):2579-84.

8. Graham S, Sullivan K, Briggs L, Goodall M, Iraci Capucinello R. A preliminary service evaluation of a personality disorder case management service. Personality and Mental Health. 2019;13(2):65-74.

9. Hassan SM, Worsley J, Nolan L, Fearon N, Ring A, Shelton J, et al. An exploration of young people’s, parent/carers’, and professionals’ experiences of a voluntary sector organisation operating a Youth Information, Advice, and Counselling (YIAC) model in a disadvantaged area. BMC health services research. 2022;22(1):1-10.

10. Giebel C, Hassan S, Harvey G, Devitt C, Harper L, Simmill-Binning C. Enabling middle-aged and older adults accessing community services to reduce social isolation: Community Connectors. Health & Social Care in the Community. 2022;30(2):e461-e8.
